# Draft Genome Sequence of Petroleum Hydrocarbon-Degrading *Pseudomonas aeruginosa* Strain PK6, Isolated from the Saurashtra Region of Gujarat, India

**DOI:** 10.1128/genomeA.00002-14

**Published:** 2014-02-06

**Authors:** P. A. Patel, V. V. Kothari, C. R. Kothari, P. R. Faldu, K. K. Domadia, C. M. Rawal, H. D. Bhimani, N. R. Parmar, N. M. Nathani, P. G. Koringa, C. G. Joshi, R. K. Kothari

**Affiliations:** a Department of Microbiology, Christ College, Rajkot, Gujarat, India; b Department of Biotechnology, Christ College, Rajkot, Gujarat, India; c Department of Biosciences, Saurashtra University, Rajkot, Gujarat, India; d Department of Animal Biotechnology, Anand Agricultural University, Anand, Gujarat, India

## Abstract

*Pseudomonas aeruginosa* strain PK6, a potential petroleum hydrocarbon-degrading soil bacterium, was isolated from a site contaminated by a petroleum hydrocarbon spill from an automobile service station in Junagadh, Gujarat, India. Here, we provide the 6.04-Mb draft genome sequence of strain PK6, which has genes encoding enzymes for potential and related metabolic pathways of the strain.

## GENOME ANNOUNCEMENT

Petroleum hydrocarbon is used as the principle source of energy in refineries and different operating companies that continuously release effluents that pollute the environment. Oil spills and effluents from these companies are major environmental pollutants that are severely affecting the ecosystem (1, 2). Thus, an ecofriendly practice is needed to develop a biological system to eradicate the pollutants. The use of microorganisms as bioremediants is a cost-effective and suitable method for the removal of petroleum hydrocarbons by their conversion into carbon dioxide and water. Microbial bioremediants include diverse bacteria, fungi, and algae that have the potential to metabolize the hydrocarbons present in crude oil (3–5).

*Pseudomonas aeruginosa* is a gammaproteobacterium that is versatile in its substrate utilization and metabolic pathways and is known for rhamnolipid production (6). *P. aeruginosa* is also reported to utilize and grow in high concentrations of crude oil (7). Whole-genome shotgun sequencing of *P. aeruginosa* strain PK6, a potential petroleum hydrocarbon-degrading soil bacterium isolated from a petroleum hydrocarbon spillage site from an automobile service station in Junagadh, Gujarat, India, was performed using the 318 Chip and 300-bp chemistry Ion Torrent PGM platform, as per the manufacturer’s instructions. When the sequence reads obtained were subjected to reference-guided assembly against the whole-genome sequence of *P. aeruginosa* M18 using the GS Reference Mapper software version 2.3, the obtained draft genome of 6,045,648 bp showed the presence of 187 contigs of >200 bp in size, with an N_50_ of 74,692 bp.

The gene annotation and screening for RNAs were performed by submitting the sequences to the Rapid Annotations using Subsystems Technology (RAST) server (8). Annotation depicted the presence of 5,954 protein-coding sequences (CDSs), of which 3,027 CDSs were assigned to one of the 553 RAST subsystems. The contigs of the genome have a 66.9% G+C content and 60 tRNAs. The genome shows about 124 CDSs related to the degradation of aromatic compounds, including genes for enzymes like catechol dioxygenase, and genes for other hydrocarbon-degrading enzymes, like alkane 1-monooxygenase and alkanesulfonate monooxygenase, were also observed. Information obtained from the whole-genome sequence about the metabolic pathways of the strain will help to reveal the genes coding for enzymes involved in exhibiting oil-degrading capabilities, which can be used in the bioremediation of crude oil-polluted sites.

### Nucleotide sequence accession number.

The sequence of *P. aeruginosa* PK6 has been deposited at GenBank under the accession no. AZBN00000000.
